# P-1589. Characterization of Blood Culture Ordering Practices at a Single Veterans Affair Medical Center

**DOI:** 10.1093/ofid/ofae631.1756

**Published:** 2025-01-29

**Authors:** Cassidy J Stegall, Tina H Dao, Nicholas Liang, Jarred Bowden, Anna Mitchell, Louis Yn, Peter Zhang, Jessica G Bennett

**Affiliations:** University of Tennessee Health Science Center College of Medicine, Memphis, TN, Memphis, Tennessee; University of Tennessee Health Science Center College of Medicine, Memphis, TN, Memphis, Tennessee; University of Tennessee Health Science Center College of Medicine, Memphis, TN, Memphis, Tennessee; Memphis VA Medical Center, Memphis, Tennessee; Lt. Col. Luke Weathers, Jr. Veterans Affairs Medical Center, Memphis, TN, Memphis, Tennessee; University of Tennessee Health Science Center College of Medicine, Memphis, TN, Memphis, Tennessee; University of Tennessee Health Science Center College of Medicine, Memphis, TN, Memphis, Tennessee; VAMC Memphis, Germantown, Tennessee

## Abstract

**Background:**

Blood culture (BCx) is the gold standard for definitive diagnosis of bacteremia and fungemia, but there is limited guidance for ordering practices. Unnecessary BCx increases the risk of false positives which can lead to increased length of stay, antibiotic use (AU), and additional testing. Diagnostic stewardship aims to optimize the use of diagnostic tests to improve patient management and outcomes. This study aimed to describe the current practice of BCx ordering at a single facility and identify areas for intervention.

**Figure:**
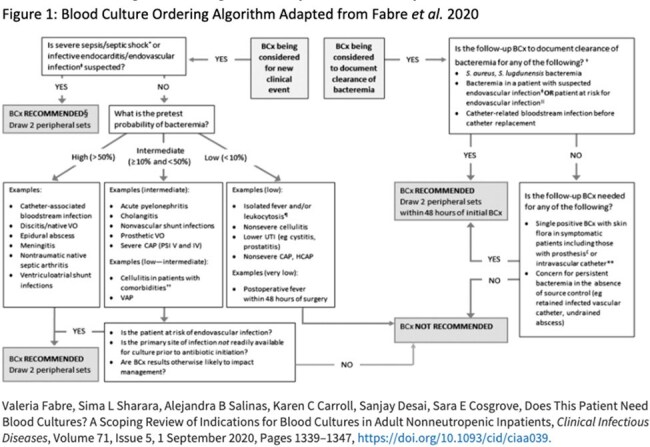

**Methods:**

This was a retrospective review of BCx ordered from 11/01/23 to 12/31/23. BCx were excluded if there was no encounter 5 days before or 2 days after BCx collection or initial BCx were drawn at another facility. Labs, vitals, BCx order date/time, ordering provider, location and results, AU and antibiotic indication were extracted from the Corporate Data Warehouse. Indications for BCx and endovascular infection risk were determined via chart review. Appropriateness of BCx was determined by an algorithm denoting low, intermediate, or high risk of bacteremia associated with an infectious disease state (Figure 1). Intermediate and high risk were deemed appropriate. Endpoints were analyzed with descriptive statistics.

**Figure:**
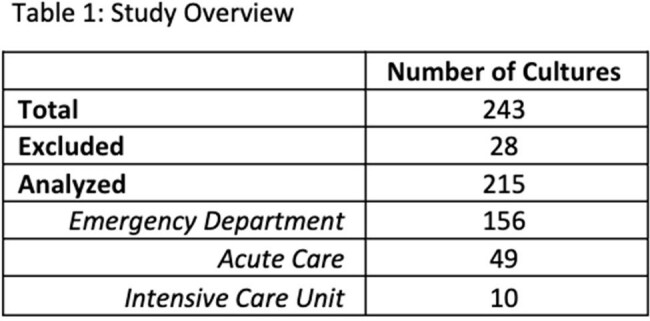

**Results:**

243 BCx were evaluated and 215 were analyzed. 156/215 (73%) were ordered in the Emergency Department (ED) (Table 1). 113/215 (53%) of BCx were appropriate with the most common indications including sepsis, intra-abdominal infection, and severe community acquired pneumonia (CAP) (Table 2). The rate of appropriateness was similar across clinical departments (Figure 2). For inappropriate BCx, 41 had no suspected infection documented, and CAP, cellulitis, and cystitis were the most documented indications (Table 2). 17/215 (8%) of BCx were positive, 6/102 in low risk, and 11/113 in intermediate/high risk group. 4/6 in the low risk were identified as contaminants.

**Figure:**
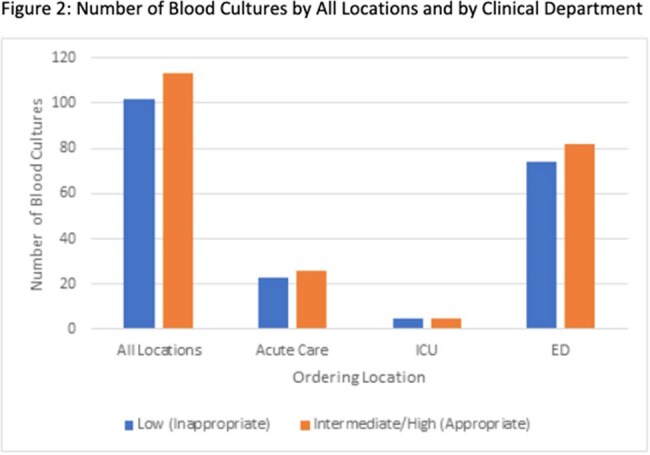

**Conclusion:**

This study found ∼50% of BCx were ordered unnecessarily, and there was both low positivity rate and false positives in this group reflecting current literature. Similar BCx ordering practices were identified across services. These findings highlight the need for intervention system wide to promote strong diagnostic stewardship and appropriate BCx ordering practices.

**Figure:**
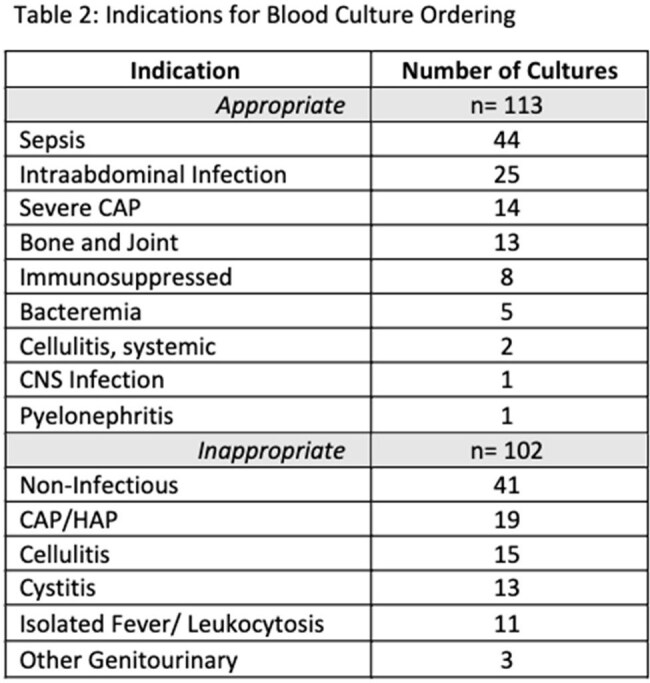

**Disclosures:**

**All Authors**: No reported disclosures

